# Single-inhaler triple vs single-inhaler dual therapy in patients with chronic obstructive pulmonary disease: a meta-analysis of randomized control trials

**DOI:** 10.1186/s12931-021-01794-w

**Published:** 2021-07-23

**Authors:** Huanyu Long, Hongxuan Xu, Jean-Paul Janssens, Yanfei Guo

**Affiliations:** 1 Department of Respiratory and Critical Care Medicine, Beijing Hospital, National Center of Gerontology; Institute of Geriatric Medicine, Chinese Academy of Medical Sciences, Beijing, China; 2 The Key Laboratory of Geriatrics, Beijing Institute of Geriatrics, Beijing Hospital, National Center of Gerontology, National Health Commission; Institute of Geriatric Medicine, Chinese Academy of Medical Sciences, Beijing, China; 3 Department of Cardiology, Beijing Hospital, National Center of Gerontology; Institute of Geriatric Medicine, Chinese Academy of Medical Sciences, Beijing, China; 4 Division of Pulmonary Diseases, Department of Medicine, Geneva University Hospitals, Geneva, Switzerland

**Keywords:** COPD, Triple therapy, Mortality, Meta-analysis

## Abstract

**Background:**

In some RCTs comparing triple therapy with dual therapy in COPD, there might be a bias resulting from the use of multiple inhaler devices. This meta-analysis included only RCTs that compared ICS/LABA/LAMA vs. LABA/LAMA or ICS/LABA using a single device.

**Methods:**

We systematically reviewed randomized controlled trials (RCTs) of single-inhaler triple therapy in patients with COPD. We searched the PubMed, MEDLINE (OvidSP), EMBASE and Cochrane Library databases to investigate the effect of single-inhaler triple therapy in COPD. The primary end points were the effect of single-inhaler triple therapy compared with single-inhaler dual therapy on all-cause mortality, the risk of acute exacerbation of COPD (AECOPD), and some safety endpoints. The Cochrane Collaboration tool was used to assess the quality of each randomized trial and the risk of bias.

**Results:**

A total of 25,171 patients suffering from COPD were recruited for the 6 studies. This meta-analysis indicated that single-inhaler triple therapy resulted in a significantly lower rate of all-cause mortality than LABA/LAMA FDC (risk ratio, 0.70; 95% CI 0.56‐0.88). Single-inhaler triple therapy reduced the risk of exacerbation and prolonged the time to first exacerbation compared with single-inhaler dual therapy. The FEV1 increased significantly more under single-inhaler triple therapy than under ICS/LABA FDC (mean difference, 103.4 ml; 95% CI 64.65‐142.15). The risk of pneumonia was, however, significantly higher with ICS/LAMA/LABA FDC than with LABA/LAMA FDC (risk ratio, 1.55; 95% CI 1.35–1.80).

**Conclusions:**

This meta-analysis suggests that single-inhaler triple therapy is effective in reducing the risk of death of any cause and of moderate or severe exacerbation in COPD patients. However, the risk of pneumonia is higher with ICS/LAMA/LABA FDC than with LABA/LAMA FDC.

*Trial registration* PROSPERO #CRD42020186726.

**Supplementary Information:**

The online version contains supplementary material available at 10.1186/s12931-021-01794-w.

## Background

Chronic obstructive pulmonary disease (COPD) is a worldwide public health challenge with a high prevalence and high morbidity and mortality rates [1, 2]. The regular administration of inhaled drugs, including long-acting beta2-agonists (LABAs), long-acting muscarinic antagonists (LAMAs), and inhaled corticosteroids (ICSs), is widely acknowledged as a major component of the treatment of COPD [3].

The Global Initiative for Obstructive Lung Disease (GOLD) management strategy recommends using ICS/LABA + LAMA in patients with persistent breathlessness, exercise limitation or persistent exacerbation, but it does not specify when to use single-inhaler triple therapy [4]. Single-inhaler triple therapy may be of benefit in patients with COPD by decreasing inhaler errors, improving adherence rates, and decreasing healthcare costs [5–7]. In some RCTs comparing triple therapy with dual therapy in COPD, there might be a bias resulting from the use of multiple inhaler devices. Single-inhaler therapy has been shown to improve lung function and health status [8, 9], but evidence of a reduction in mortality with single-inhaler triple therapy versus single-inhaler dual therapy has not been well documented in previous meta-analyses.

We therefore performed this systematic review to determine the effect of ICS/LABA/LAMA compared with LABA/LAMA or ICS/LABA using a single device on the risk of mortality and exacerbation and on other relevant outcomes in patients with COPD.

## Methods

### Search strategy

This meta-analysis followed the guidelines of the Preferred Reporting Items for Systematic Reviews and Meta-analyses (PRISMA) statement [10]. This study was prospectively registered in Prospero (CRD42020186726).

We used the following search terms in the PubMed, MEDLINE (OvidSP), EMBASE and Cochrane Library databases to identify studies published up to May 15, 2021: “chronic obstructive pulmonary disease”, “triple”, “long-acting antimuscarinics”, “long-acting beta-2 agonists” or “inhaled corticosteroids”. The “Patients, Intervention, Control, and Outcome” (PICO) framework was utilized to improve the relevance of the search results, as previously described [11]. The patients included were those with “COPD”, the intervention was “single-inhaler triple therapy (LABA/LAMA/ICS)”, the control arm was “single-inhaler dual therapy (ICS/LABA or LABA/LAMA)”, and outcomes included “death, risk of moderate or severe exacerbation, time to exacerbation, lung function, health-related quality of life and safety profile” (see Additional file 1: Table S1). The search strategy was performed as shown in Additional file 1: Table S2.

### Study selection and data extraction

Data were independently extracted by two reviewers. Any difference in opinion about eligibility was resolved through consensus. We collected information from each randomized trial about study features (title, year, author, study design and duration of follow-up, etc.), participants (mean age, sex, current smoker, etc.), interventions (control therapy and inhaler type, intervention therapy and inhaler type), and outcomes (death, moderate or severe exacerbation, time to first exacerbation, mean change in FEV1, SGRQ (St. George Respiratory Questionnaire) score, adverse events, serious adverse events, cardiovascular events and pneumonia events). When data could not be extracted from the published reports, we extrapolated them from the supplementary material.

### Quality score and risk-of-bias assessment

Cochrane's Collaboration tool was used to assess the quality of each randomized trial and the risk of bias. We analysed included trials for allocation concealment, random sequence generation, blinding of the outcome assessment, incomplete outcome data, selective reporting, blinding of the participants and personnel, and other biases.

### Data synthesis and statistical analysis

We used RevMan 5.3 software for all statistical analyses. The degree of heterogeneity among RCTs was evaluated with the Q test and I^2^ statistic. I^2^ values ≥ 50% were considered to represent significant heterogeneity, in which case a random-effects model was applied. We combined continuous data using the inverse-variance test for the risk ratio, hazard ratio, rate ratio, and mean difference with 95% confidence intervals (95% CIs) and combined dichotomous data using the Mantel–Haenszel test for risk ratios with 95% confidence intervals.

Due to expected clinical heterogeneity, we evaluated single-inhaler triple therapy vs. LABA/LAMA or ICS/LABA FDC. The Cochran Q test for subgroup differences was used to determine the significance of subgroup interactions for all outcomes.

## Results

**Fig. 1:**
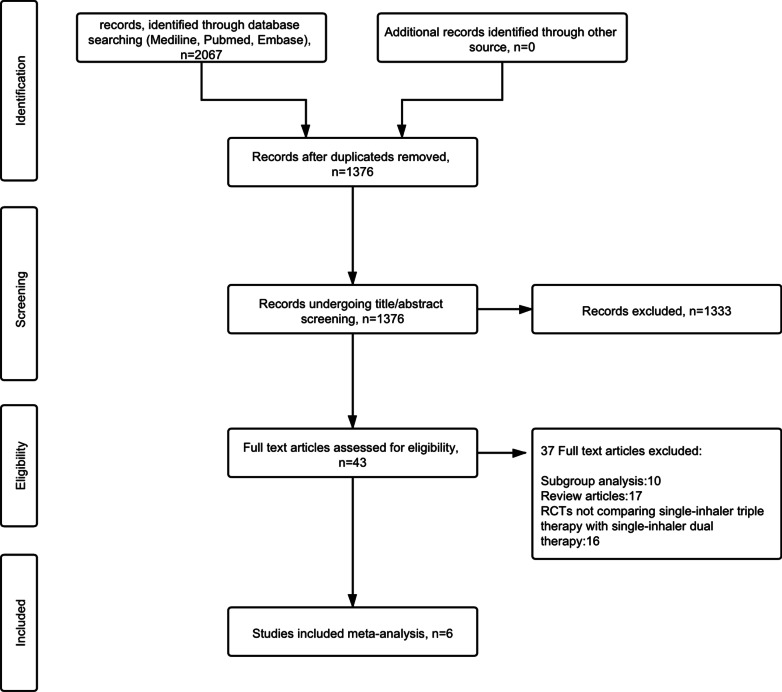
Flow diagram of the study selection process. *RCTs* randomized controlled trials

**Fig. 2:**
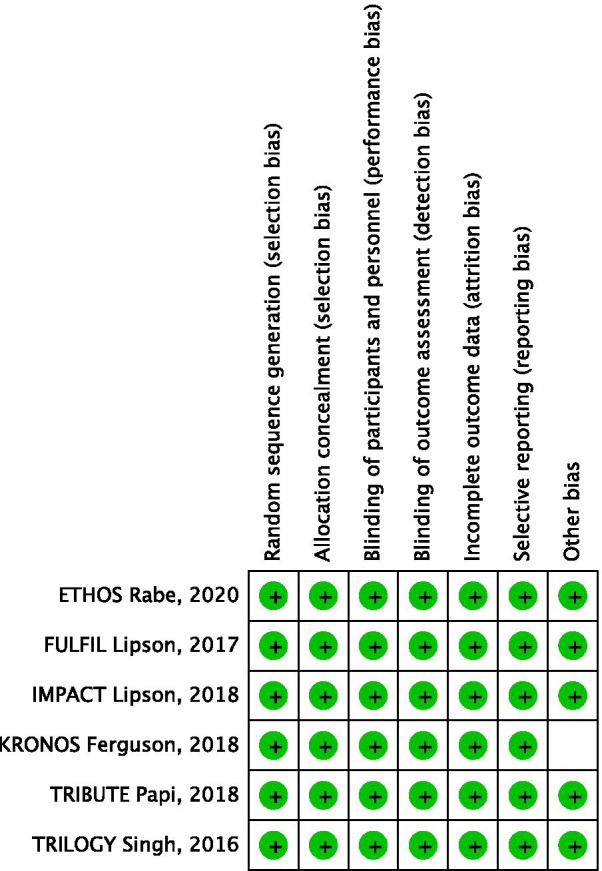
Risk of bias summary for the included RCTs. *RCTs* randomized controlled trials

We obtained 2,067 articles from our initial search, and 43 additional articles were identified through manual searches. At the end of the selection process, 6 RCTs [12–17] were included in this meta-analysis. A flow diagram of the study selection process is shown in Fig. 1. A total of 25,171 COPD patients were recruited for these 6 studies: 11,420 patients were treated with single-inhaler triple therapy, 5,588 patients were treated with LABA/LAMA FDC, and 8,163 patients were treated with ICS/LABA FDC. A summary of the relevant studies and patient characteristics is provided in Tables 1 and 2. The risk of bias of the included studies is detailed in Fig. 2.

**Table 1:** Details of included RCTs

Number	Study	Study design	Duration of follow-up	Inclusion criteria	Exclusion criteria	Drugs, Doses (μg), Regimen, Device	No of patients
1	TRILOGY Singh, 2016	A randomized, parallel group, double-blind, active-controlled study	52 weeks	FEV1 < 50% predicted, moderate or severe COPD exacerbation in the previous 12 months ≥ 1, CAT score ≥ 10, BDI score ≤ 10	Asthma, history of allergic rhinitis; clinically significant cardiovascular conditions	BDP/FOR/GLY (100/6/12.5) bid MDI	687
						BDP/FOR (100/6) bid MDI	680
2	FULFIL Lipson,2017	A phase 3, randomized, double-blind, double-dummy, parallel-group, multicentre study	24 weeks	FEV1 < 50% predicted and CAT score ≥ 10; 50% ≤ FEV1 < 80% predicted and CAT score ≥ 10 or moderate COPD exacerbation in the previous 12 months ≥ 2 or severe COPD exacerbation in the previous 12 months ≥ 1	Asthma, unresolved pneumonia, severe COPD exacerbation	FF/UMEC/VI (100/62.5/25) od DPI	911
						BUD/FOR (400/12) bid DPI	899
3	IMPACT Lipson,2018	A phase 3, randomized, double-blind, parallel-group, multicentre trial	52 weeks	FEV1 < 50% predicted and moderate or severe COPD exacerbation in the previous 12 months ≥ 1; 50% ≤ FEV1 < 80% predicted and moderate COPD exacerbation in the previous 12 months ≥ 2 or severe COPD exacerbation in the previous 12 months ≥ 1	Not reported	FF/UMEC/VI (100/62.5/25) od DPI	4151
						FF/VI (100/25) od DPI	4134
						UMEC/VI (62.5/25) od DPI	2070
4	KRONOS Ferguson,2018	A randomized, double-blind, parallel-group, phase 3 randomized controlled trial	24 weeks	25% ≤ FEV1 < 80% predicted; CAT score ≥ 10. Patients were not required to have had a COPD exacerbation within the preceding year	Asthma, diagnosis of any respiratory disease	BUD/GLY/FOR (320/18/9.6) bid MDI	639
						GLY/FOR (18/9.6) bid MDI	625
						BUD/FOR (320/9.6) bid MDI	314
5	TRIBUTE Papi,2018	A randomized, parallel-group, double-blind, double-dummy, active-controlled phase 3b study	52 weeks	FEV1 < 50%, a moderate or severe COPD exacerbation in the previous 12 months ≥ 1, CAT score ≥ 10	Asthma; clinically significant cardiovascular disorders	BDP/FOR/GLY (100/6/10) bid MDI	764
						IND/GLY (85/43) od DPI	768
6	ETHOS Rabe,2020	A phase 3, randomized, double-blind, parallel-group, multicentre trial	52 weeks	FEV1 < 50% predicted and moderate or severe COPD exacerbation in the previous 12 months ≥ 1; FEV1 ≥ 50% predicted and moderate COPD exacerbation in the previous 12 months ≥ 2 or severe COPD exacerbation in the previous 12 months ≥ 1, CAT ≥ 10	Current diagnosis of asthma	BUD/GLY/FOR (320/18/9.6) bid MDI	2144
						BUD/GLY/FOR (160/18/9.6) bid MDI	2124
						BUD/FOR (320/9.6) bid MDI	2136
						GLY/FOR (18/9.6) bid MDI	2125

BDP/FOR/GLY: beclomethasone dipropionate/formoterol fumarate/glycopyrronium bromide; BUD/GLY/FOR: budesonide/glycopyrronium bromide/formoterol fumarate; FF/UMEC/VI: fluticasone furoate/umeclidinium/vilanterol; BDP/FOR: beclomethasone dipropionate/formoterol fumarate; BUD/FOR: budesonide/formoterol fumarate; UMEC/VI: umeclidinium bromide/vilanterol; GLY/FOR: glycopyrronium bromide/formoterol fumarate; IND/GLY: indacaterol/glycopyrronium bromide; OD: once daily; BID: twice daily; MDI: metered-dose inhaler formulation; DPI: dry powder inhaler formulation; FEV1: forced expiratory volume in 1 s; BDI: Baseline Dyspnea Index; CAT: COPD assessment test; RCT: Randomized controlled trial

**Table 2:** Patient baseline characteristics

Number	Study	Drugs, Doses (μg), Regimen, Device	No of patients	Age means (SD)	Male (%)	Current smoker (%)	Postbronchodilator FEV1, % Predicted (SD)	Moderate/severe COPD exacerbation in previous 12 months, n (%) 0	Moderate/severe COPD exacerbation in previous 12 months, n (%) 1	Moderate/severe COPD exacerbation in previous 12 months, n (%) ≥ 2
1	TRILOGY Singh, 2016	BDP/FOR/GLY (100/6/12.5) bid MDI	687	63.3 (7.9)	74	47	36.9 (8.4)	NA	NA	NA
		BDP/FOR (100/6) bid MDI	680	63.8 (8.2)	77	47	36.2 (8.6)	NA	NA	NA
2	FULFIL Lipson,2017	FF/UMEC/VI (100/62.5/25) od DPI	911	64.2 (8.56)	74	44	45.5 (12.97)	34	28	38
		BUD/FOR (400/12) bid DPI	899	63.7 (8.71)	74	44	45.1 (13.64)	35	28	37
3	IMPACT Lipson,2018	FF/UMEC/VI (100/62.5/25) od DPI	4151	65.3 (8.2)	67	35	45.7 (15.0)	< 1	45	55
		FF/VI (100/25) od DPI	4134	65.3 (8.3)	66	34	45.5 (14.8)	< 1	46	54
		UMEC/VI (62.5/25) od DPI	2070	65.2 (8.3)	66	35	45.4 (14.7)	< 1	45	55
4	KRONOS Ferguson,2018	BUD/GLY/FOR (320/18/9.6) bid MDI	639	64.9 (7.8)	72	40.1	50.2 (14.3)	73.4	19.6	7
		GLY/FOR (18/9.6) bid MDI	625	65.1 (7.7)	68.8	41.1	50.2 (13.8)	75.7	17.3	7
		BUD/FOR (320/9.6) bid MDI	314	65.2 (7.2)	71.3	36.6	50 (14)	74.8	19.4	5.7
5	TRIBUTE Papi,2018	BDP/FOR/GLY (100/6/10) bid MDI	764	64.4 (7.7)	72	46	36.4 (8.0)	NA	80	20
		IND/GLY (85/43) od DPI	768	64.5 (7.7)	72	43	36.4 (8.1)	NA	82	18
6	ETHOS Rabe,2020	BUD/GLY/FOR (320/18/9.6) bid MDI	2144	64.6 (7.6)	59	42.6	43.6 (10.3)	0.1	44	55.9
		BUD/GLY/FOR (160/18/9.6) bid MDI	2124	64.6 (7.6)	61.2	40.8	43.1 (10.4)	0.1	43.9	56
		BUD/FOR (320/9.6) bid MDI	2136	64.6 (7.6)	60	40.5	43.4 (10.4)	0.1	42.8	57.1
		GLY/FOR (18/9.6) bid MDI	2125	64.8 (7.6)	58.7	40.4	43.5 (10.2)	0.1	42.8	57.1

BDP/FOR/GLY: beclomethasone dipropionate/formoterol fumarate/glycopyrronium bromide; BUD/GLY/FOR: budesonide/glycopyrronium bromide/formoterol fumarate; FF/UMEC/VI: fluticasone furoate/umeclidinium/vilanterol; BDP/FOR: beclomethasone dipropionate/formoterol fumarate; BUD/FOR: budesonide/formoterol fumarate; UMEC/VI: umeclidinium bromide/vilanterol; GLY/FOR: glycopyrronium bromide/formoterol fumarate; IND/GLY: indacaterol/glycopyrronium bromide; OD: once daily; BID: twice daily; MDI: metered-dose inhaler formulation; DPI: dry powder inhaler formulation; SD: Standard deviation; NA: Not applicable

### Efficacy endpoints

**Fig. 3:**
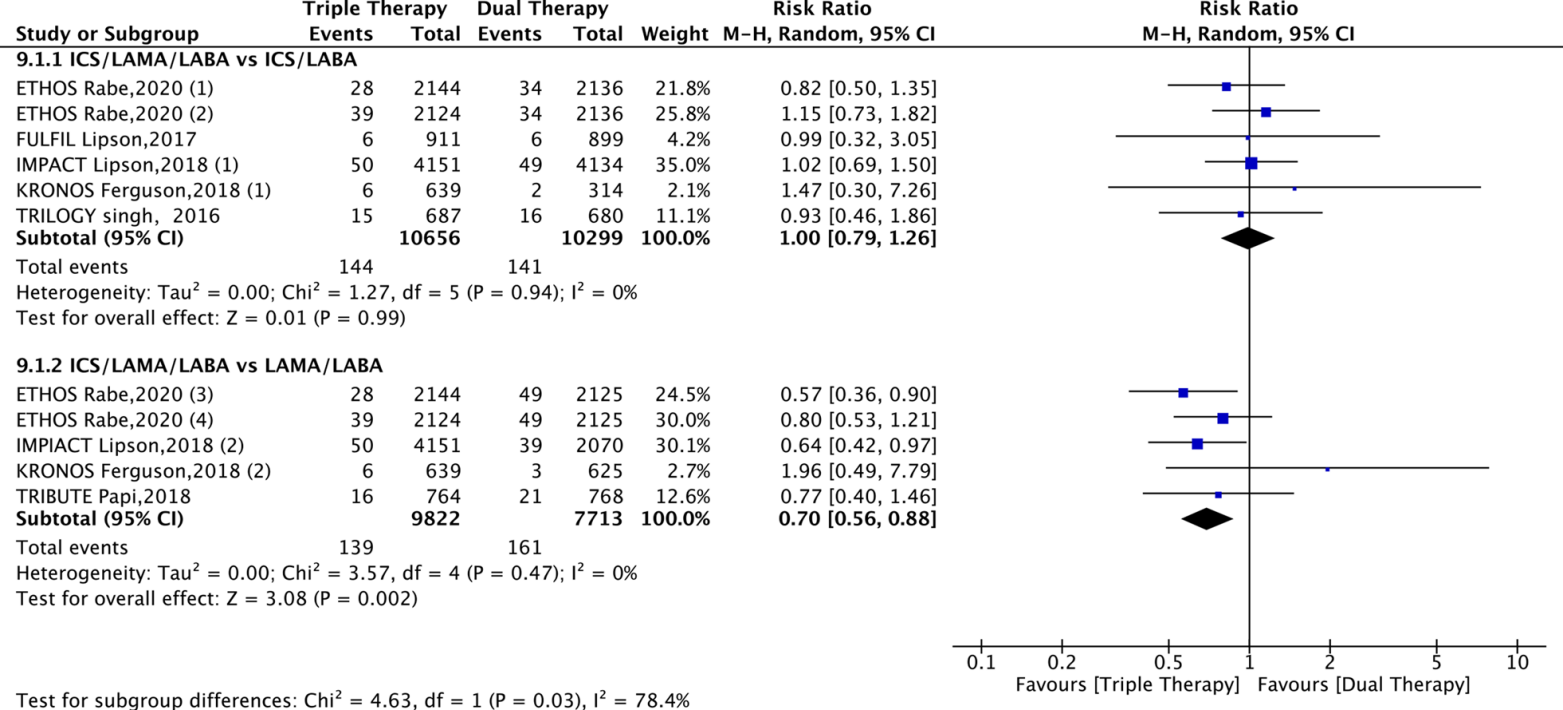
Forest plot for all-cause mortality. *ICSs* inhaled corticosteroids, *LABA* long-acting beta2-agonist, *LAMA* long-acting muscarinic antagonist. ETHOS Rabe, 2020 (1): BUD/GLY/FOR (320/18/9.6) vs. BUD/FOR (320/9.6); ETHOS Rabe, 2020 (2): BUD/GLY/FOR (160/18/9.6) vs. BUD/FOR (320/9.6); ETHOS Rabe, 2020 (3): BUD/GLY/FOR (320/18/9.6) vs. GLY/FOR (18/9.6); ETHOS Rabe, 2020 (4): BUD/GLY/FOR (160/18/9.6) vs. GLY/FOR (18/9.6); IMPACT Lipson, 2018 (1): FF/UMEC/VI (100/62.5/25) vs. FF/VI (100/25); IMPACT Lipson, 2018 (2): FF/UMEC/VI (100/62.5/25) vs. UMEC/VI (62.5/25); KRONOS Ferguson, 2018 (1): BUD/GLY/FOR (320/18/9.6) vs. BUD/FOR (320/9.6); KRONOS Ferguson, 2018 (2): BUD/GLY/FOR (320/18/9.6) vs. GLY/FOR (18/9.6). BUD/GLY/FOR: budesonide/glycopyrronium bromide/formoterol fumarate; FF/UMEC/VI: fluticasone furoate/umeclidinium/vilanterol; BUD/FOR: budesonide/formoterol fumarate; UMEC/VI: umeclidinium bromide/vilanterol; GLY/FOR: glycopyrronium bromide/formoterol fumarate

This meta-analysis suggested that compared with patients receiving LABA/LAMA FDC, those receiving ICS/LAMA/LABA FDC had a significantly lower mortality rate (risk ratio, 0.70; 95% CI 0.56–0.88; *P* < 0.01; I^2^ = 0%); however, no significant difference was found between ICS/LAMA/LABA FDC and ICS/LABA FDC (risk ratio, 1.00; 95% CI 0.79–1.26; *P* > 0.05; I^2^ = 0%) (Fig. 3).

**Fig. 4:**
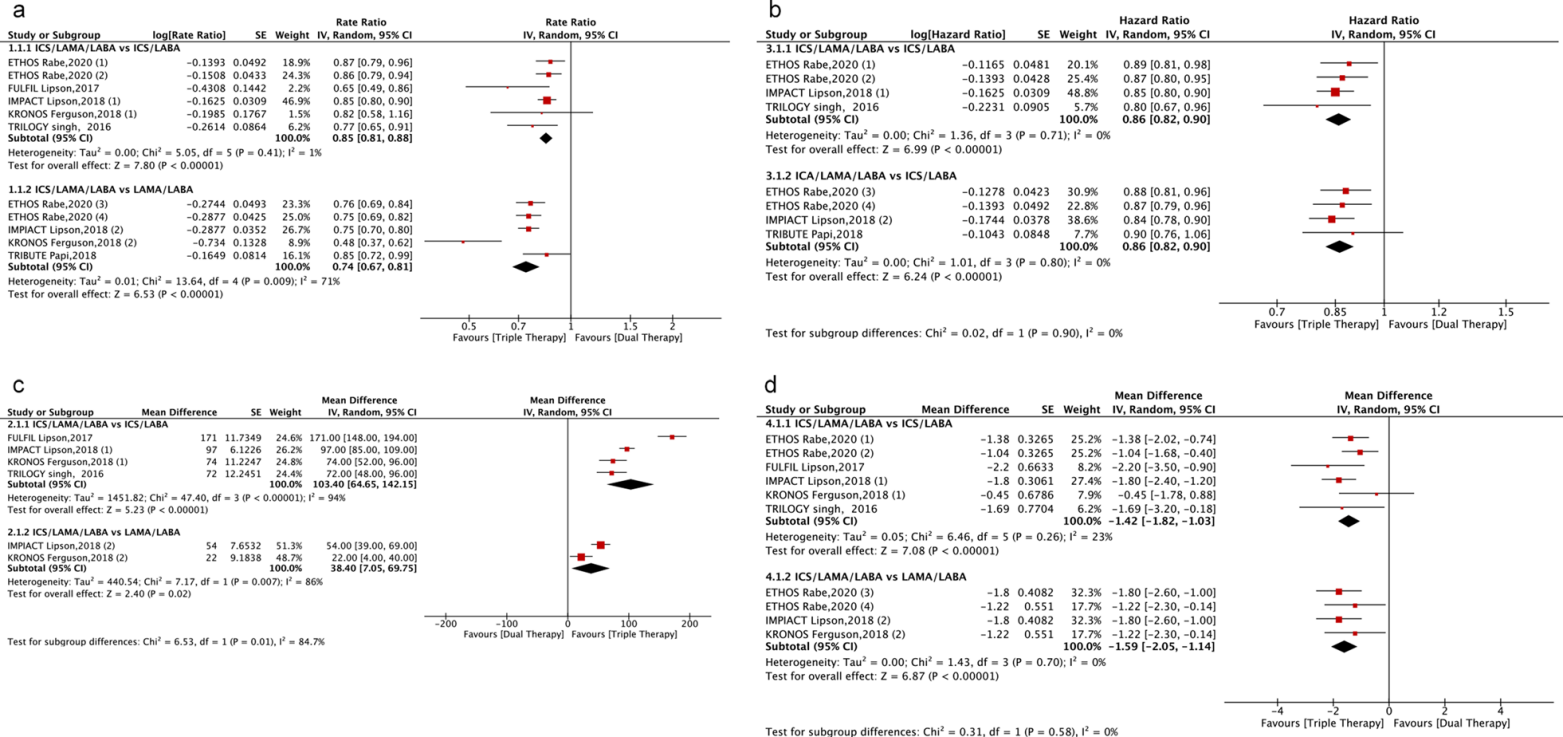
Forest plot for efficacy endpoints. Forest plot of the impact of single-inhaler triple therapy vs. single-inhaler dual therapy (LABA/LAMA or ICS/LABA FDC) on **a** moderate‐to‐severe COPD exacerbation; **b** time to first exacerbation; **c** mean difference in the forced expiratory volume in 1 s (FEV1); **d** mean difference in the St. George's Respiratory Questionnaire (SGRQ) score. *ICSs* inhaled corticosteroids, *LABA* long-acting beta2-agonist, *LAMA* long-acting muscarinic antagonist, ETHOS Rabe, 2020 (1): BUD/GLY/FOR (320/18/9.6) vs. BUD/FOR (320/9.6); ETHOS Rabe, 2020 (2): BUD/GLY/FOR (160/18/9.6) vs. BUD/FOR (320/9.6); ETHOS Rabe, 2020 (3): BUD/GLY/FOR (320/18/9.6) vs. GLY/FOR (18/9.6); ETHOS Rabe, 2020 (4): BUD/GLY/FOR (160/18/9.6) vs. GLY/FOR (18/9.6); IMPACT Lipson, 2018 (1): FF/UMEC/VI (100/62.5/25) vs. FF/VI (100/25); IMPACT Lipson, 2018 (2): FF/UMEC/VI (100/62.5/25) vs. UMEC/VI (62.5/25); KRONOS Ferguson, 2018 (1): BUD/GLY/FOR (320/18/9.6) vs. BUD/FOR (320/9.6); KRONOS Ferguson, 2018 (2): BUD/GLY/FOR (320/18/9.6) vs. GLY/FOR (18/9.6). BUD/GLY/FOR: budesonide/glycopyrronium bromide/formoterol fumarate; FF/UMEC/VI: fluticasone furoate/umeclidinium/vilanterol; BUD/FOR: budesonide/formoterol fumarate; UMEC/VI: umeclidinium bromide/vilanterol; GLY/FOR: glycopyrronium bromide/formoterol fumarate

The use of single-inhaler triple therapy was associated with a significant decrease in the risk of moderate or severe COPD exacerbation compared with ICS/LABA FDC (rate ratio, 0.85; 95% CI 0.81–0.88; *P* < 0.01; I^2^ = 1%) and LABA/LAMA FDC (rate ratio, 0.74; 95% CI 0.67–0.81; *P* < 0.01; I^2^ = 71%) (Fig. 4).

The time to first exacerbation was significantly longer in patients under single-inhaler triple therapy than in those on ICS/LABA FDC (hazard ratio, 0.86; 95% CI 0.8–0.90; *P* < 0.01; I^2^ = 1%) and LABA/LAMA FDC (hazard ratio, 0.86; 95% CI 0.82–0.90; *P* < 0.01; I^2^ = 0%) (Fig. 4).

The FEV1 (trough FEV1 compared to baseline, ml) increased significantly more under single-inhaler triple therapy than under ICS/LABA FDC (mean difference, 103.4 ml; 95% CI 64.65–142.15; *P* < 0.01; I^2^ = 94%) or LABA/LAMA FDC (mean difference, 38.40 ml; 95% CI 7.05–69.75; *P* < 0.05; I^2^ = 86%) (Fig. 4).

Improvement in health-related quality of life (HRQoL: SGRQ total score) was significantly higher with single-inhaler triple therapy than with ICS/LABA FDC (mean difference, -1.42; 95% CI − 1.82 to − 1.03; *P* < 0.01; I^2^ = 23%) or LABA/LAMA FDC (mean difference, − 1.59; 95% CI − 2.05 to − 1.14; *P* < 0.01; I^2^ = 0%) (Fig. 4).

### Safety endpoints

**Fig. 5:**
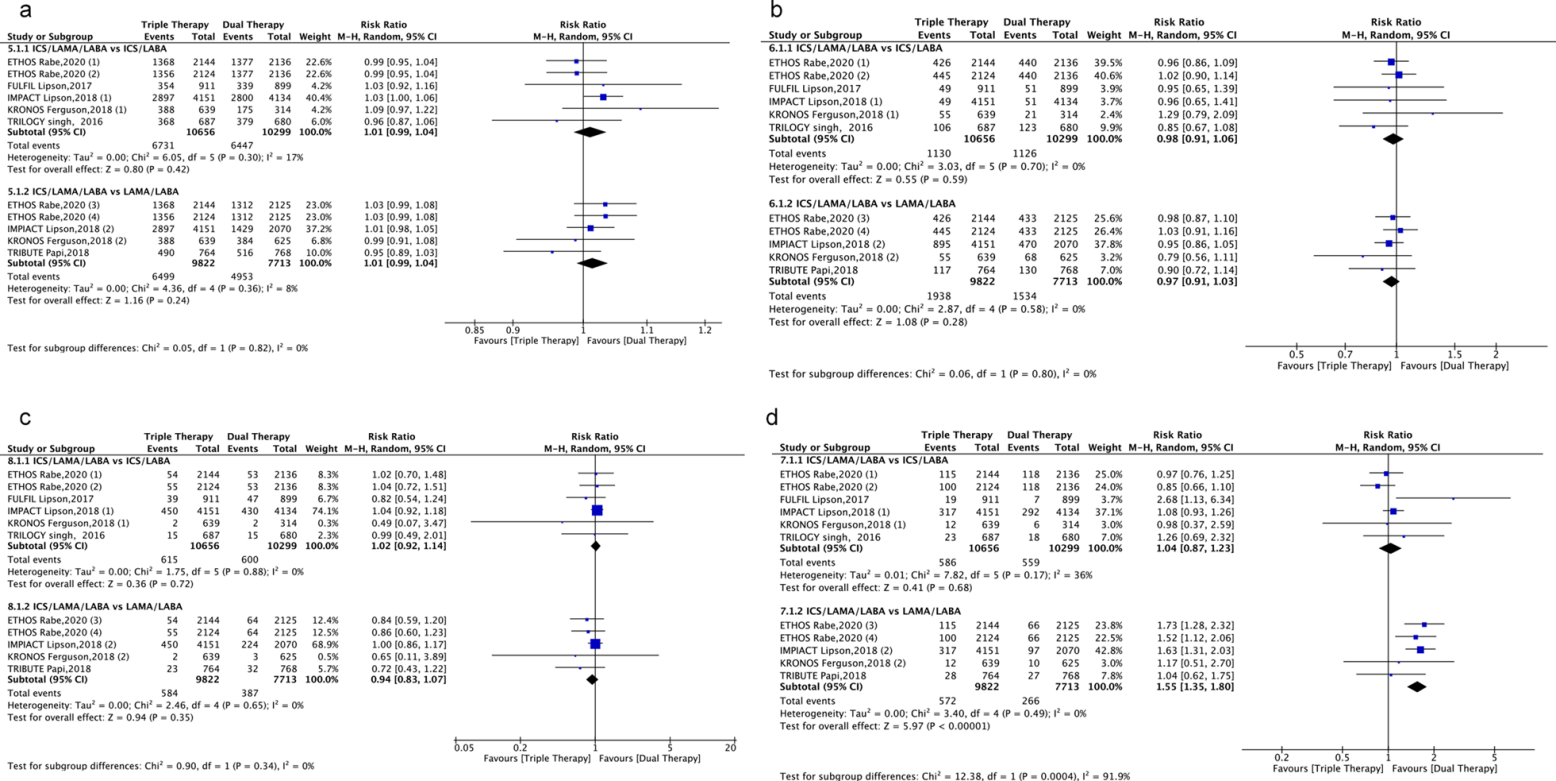
Forest plot for safety endpoints. Single-inhaler triple therapy vs. single-inhaler dual therapy (LABA/LAMA or ICS/LABA FDC) on the risk of adverse events (AEs) and serious adverse events (**a**, **b**) and the risk of cardiovascular events and pneumonia (**c**, **d**) in COPD patients. ICSs: Inhaled corticosteroids; LABA: Long-acting beta2-agonist; LAMA: Long-acting muscarinic antagonist; ETHOS Rabe, 2020 (1): BUD/GLY/FOR (320/18/9.6) vs. BUD/FOR (320/9.6); ETHOS Rabe, 2020 (2): BUD/GLY/FOR (160/18/9.6) vs. BUD/FOR (320/9.6); ETHOS Rabe, 2020 (3): BUD/GLY/FOR (320/18/9.6) vs. GLY/FOR (18/9.6); ETHOS Rabe, 2020 (4): BUD/GLY/FOR (160/18/9.6) vs. GLY/FOR (18/9.6); IMPACT Lipson, 2018 (1): FF/UMEC/VI (100/62.5/25) vs. FF/VI (100/25); IMPACT Lipson, 2018 (2): FF/UMEC/VI (100/62.5/25) vs. UMEC/VI (62.5/25); KRONOS Ferguson, 2018 (1): BUD/GLY/FOR (320/18/9.6) vs. BUD/FOR (320/9.6); KRONOS Ferguson, 2018 (2): BUD/GLY/FOR (320/18/9.6) vs. GLY/FOR (18/9.6). BUD/GLY/FOR: budesonide/glycopyrronium bromide/formoterol fumarate; FF/UMEC/VI: fluticasone furoate/umeclidinium/vilanterol; BUD/FOR: budesonide/formoterol fumarate; UMEC/VI: umeclidinium bromide/vilanterol; GLY/FOR: glycopyrronium bromide/formoterol fumarate

Single-inhaler triple therapy was not associated with an increase in adverse events (*P* > 0.05) (Fig. 5) or serious adverse events (*P* > 0.05) when compared with single-inhaler dual therapy. This was also the case for cardiovascular events (*P* > 0.05) (Fig. 5). The risk of pneumonia did not differ between ICS/LAMA/LABA FDC and ICS/LABA FDC (risk ratio, 1.04; 95% CI 0.87− 1.23; *P* > 0.05; I^2^ = 36%), but the use of ICS/LAMA/LABA FDC was associated with a significant increase in the risk of pneumonia compared with LABA/LAMA FDC (risk ratio, 1.55; 95% CI 1.35− 1.80; *P* < 0.01; I^2^ = 0%) (Fig. 5).

## Discussion

This systematic review aimed to investigate the long‐term effects (≥ 24 weeks) of single-inhaler triple therapy compared with single-inhaler dual therapy (ICS/LABA or LABA/LAMA FDC) for the treatment of COPD. Our results suggest that ICS/LAMA/LABA FDC was more effective in reducing all-cause mortality than LABA/LAMA FDC and more effective in reducing the risk of moderate or severe COPD exacerbation and prolonging the time to first exacerbation than ICS/LABA or LABA/LAMA FDC. Furthermore, single-inhaler triple therapy had a significantly higher impact on lung function (trough FEV1) than ICS/LABA FDC. However, the risk of pneumonia was significantly higher with ICS/LAMA/LABA FDC than with LABA/LAMA FDC.

Two recent meta-analyses showed that single-inhaler triple therapy was more effective in reducing acute exacerbation and improving lung function than single-inhaler dual therapy [8, 18]. However, to the best of our knowledge, this meta-analysis is the first to show a reduction in all-cause mortality in stable COPD with triple therapy vs LABA/LAMA using a single device.

The goal of COPD management is to decrease the risk of exacerbation and mortality [1]. Exacerbation is a major determinant of the patient's health status and a strong predictor of mortality [19, 20]. Mortality increases with the frequency of severe exacerbation episodes, particularly if these episodes require admission to the hospital [21]. Our study shows that ICS/LAMA/LABA FDC reduced all-cause mortality compared with LABA/LAMA FDC, but there was no significant difference compared with ICS/LABA FDC. In the IMPACT [16] (FF/UMEC/VI vs. UMEC/VI) and ETHOS [17] (BUD/GLY/FOR (320 μg of budesonide) vs. GLY/FOR) studies, the risk of death from any cause was reduced by 29% and 46%, respectively. The all-cause mortality reduction by ICS/LAMA/LABA FDC may be due to the reduction in the total number of exacerbation episodes, which can improve the patient’s health status and decrease the rate of hospitalization [22, 23], thus decreasing the associated morbidity and mortality rates in COPD patients. The present study shows that compared with single-inhaler dual therapy, single-inhaler triple therapy significantly reduced the frequency of moderate and severe exacerbation episodes. Our results are consistent with the findings of the most recent meta-analyses [9, 24]. In the ETHOS [17] study, which compared GLY/FOR to BUD/GLY/FOR (320 μg of budesonide), the frequency of moderate and severe exacerbation episodes decreased by 24% with BUD/GLY/FOR vs. GLY/FOR. The IMPACT [16] study showed a 25% decrease in the COPD exacerbation rate and a 34% reduction in the number of COPD hospitalizations when comparing FF/UMEC/VI to UMEC/VI. The risk of pneumonia was higher for ICS/LAMA/LABA FDC than for LABA/LAMA FDC. This is consistent with previous findings [8, 18]. However, the risk of pneumonia was unlikely to result in an increased risk of all-cause mortality in our study. Previous studies have found that the use of ICSs does not increase the rate of pneumonia-related mortality [25, 26]. Mammen and colleagues suggested that the incidence of AECOPD is greater than the incidence of pneumonia at baseline. The reduction in the COPD exacerbation rate is likely to be more clinically important than the increase in the risk of pneumonia with the use of triple therapy versus dual LABA/LAMA therapy [27].

Although investigators have found statistically significant differences in important outcomes between treatment groups, these results must also be interpreted with caution, as the differences found may not be clinically meaningful. According to Jones 2013 [28] and Bateman 2014 [29], the consensus on the minimum clinically important difference (MCID) for the trough FEV1 is 60 mL, and that for the SGRQ score is 4 units. Thus, the benefit of single-inhaler triple therapy compared with ICS/LABA FDC on the trough FEV1 (103 ml) exceeded the MCID. In terms of HRQoL (SGRQ score), differences between single-inhaler triple therapy and single-inhaler dual therapy were statistically significant but below the accepted MCID. Further trials evaluating the relationship between HRQoL and the benefits of single-inhaler triple therapy are warranted.

There were differences in study designs and populations that could contribute to heterogeneity in our meta-analysis. First, the FULFIL [13] and KRONOS [14] studies were of only 24 weeks in duration and limited in their reporting of health outcomes. Second, the severity of COPD differed among the included RCTs, particularly in relation to prior exacerbation history. Inclusion in the KRONOS study [14] did not require having an exacerbation episode within the preceding year, thus potentially including patients for which triple therapy was not formally recommended according to the recent GOLD update. Finally, the TRILOGY [12] and RIBUTE [15] studies excluded patients with significant cardiovascular conditions (including but not limited to unstable ischaemic heart disease, NYHA class III/IV heart failure, left ventricular failure, and acute myocardial infarction), while other studies did not mention these exclusion criteria. Differences in the exclusion criteria may affect the mortality rates, and single-inhaler triple therapy may have direct or indirect effects on cardiovascular comorbidity in COPD patients and thus on non-respiratory fatal events [30].

Although our meta-analysis revealed the superiority of single-inhaler triple therapy over single-inhaler dual therapy in patients with COPD, we were not able to assess its effects based on variations in the eosinophil count. In the ETHOS study, the annual rate of moderate or severe exacerbation was lower with single-inhaler triple therapy than with either single-inhaler dual therapy, regardless of the eosinophil count (< 150 and ≥ 150 cells per cubic millimetre) [17], a finding consistent with the IMPACT study [16]. A meta-analysis suggested that in non-eosinophilic subjects single-inhaler triple therapy was also superior to both LABA/LAMA and ICS/LABA FDC in reducing COPD exacerbation [18].

The GOLD guidelines recommend that triple therapy be considered for the most severe COPD patients [3]. Patients using multiple devices are more likely to have an inappropriate inhalation technique [31]. Additionally, previous research has shown that COPD patients have a lower rate of adherence to treatment with multiple-inhaler therapy than single-inhaler therapy [32, 33]. Single-inhaler therapy is simpler and thus may lead to better compliance and improved clinical outcomes in COPD patients [34] and therefore decrease healthcare resource utilization [7, 35]. If these outcomes are achieved without increasing costs, this may reduce the economic and healthcare resource burden [6].

Our research has a few limitations. First, some of the included RCTs were performed over only 24 weeks, thus limiting their relevance for outcomes such as all-cause mortality. Second, the analysed RCTs, despite having similar criteria for eligibility, did have some differences in the inclusion criteria, which may impact the severity and rate of complications. Further studies are needed to determine whether any specific subgroup of COPD patients is more likely to benefit from single-inhaler triple therapy. Finally, patients were undergoing dual or triple therapy at baseline; it is therefore unclear whether the abrupt discontinuation of previous medication could have contributed to these results.

## Conclusion

Our meta-analysis suggests a beneficial effect of single-inhaler triple therapy in terms of mortality, frequency of moderate or severe COPD exacerbation episodes, and lung function in symptomatic COPD patients. However, ICS/LAMA/LABA FDC is associated with an increased risk of pneumonia compared to LABA/LAMA FDC.

## Supplementary Information

**Additional file 1: Table S1.** PICO question formulation. **Table S2.** Search strategy.

## Data Availability

All data used or analysed during this study are included in this published article.

## Abbreviations

AE: Adverse event
AECOPD: Acute exacerbation of COPD
BDI: Baseline Dyspnoea Index
BDP: Beclomethasone dipropionate
BID: Twice daily
BUD: Budesonide
CAT: COPD assessment test
CI: Confidence interval
COPD: Chronic obstructive pulmonary disease
DPI: Dry powder inhaler formulation
ETHOS: Efficacy and Safety of Triple Therapy in Obstructive Lung Disease
FDC: Fixed-dose combination
FEV1: Forced expiratory volume in one second
FF: Fluticasone furoate
FOR: Formoterol fumarate
FULFIL: Lung Function and Quality of Life Assessment in Chronic Obstructive Pulmonary Disease with Closed Triple Therapy
GLY: Glycopyrronium bromide
GOLD: Global Initiative for Chronic Obstructive Lung Disease
HRQoL: Health-related quality of life
ICSs: Inhaled corticosteroids
IMPACT: Informing the Pathway of COPD Treatment
IND: Indacaterol
LABA: Long-acting beta2-agonist
LAMA: Long-acting muscarinic antagonist
MCID: Minimum clinically important difference
MDI: Metred-dose inhaler formulation
NA: Not applicable
OD: Once daily
PRISMA: Preferred Reporting Items for Systematic Reviews and Meta-analyses
RCT: Randomized controlled trial
SAE: Serious adverse event
SD: Standard deviation
SGRQ: St. George’s Respiratory Questionnaire
TIO: Tiotropium
UMEC: Umeclidinium
VI: Vilanterol
