# Supplementary material for: Single-inhaler triple vs single-inhaler dual therapy in patients with chronic obstructive pulmonary disease: a meta-analysis of randomized control trials
Source: Respir Res. 2021 Jul 23;22:209. doi: 10.1186/s12931-021-01794-w (PMC8299655; doi:10.1186/s12931-021-01794-w)
Supplement: Supplementary file 1 — Additional file 1: Table S1. PICO question formulation. Table S2. Search strategy. [file 12931_2021_1794_MOESM1_ESM.pdf]

Supplementary Table 1. PICO question formulation

Questions	Description
Population	Chronic Obstructive Lung Disease
Interventions	single-inhaler triple therapy with ICS/LABA/LAMA
Comparator	single-inhaler dual therapy with ICS/LABA or LABA/LAMA
Outcomes	frequency/severity of exacerbations
	death
	lung function
	quality of life
	adverse events

ICS: inhaled corticosteroids; LABA: long-acting beta-2 agonist, LAMA: long-acting muscarinic antagonist; PICO: Population, Interventions, Comparator, Outcomes.

Supplementary Table 2. Search strategy

1	"randomized controlled trial".pt.
2	(random$ or placebo$ or single blind$ or double blind$ or triple blind$).ti,ab.
3	(retraction of publication or retracted publication).pt.
4	or/1-3
5	(animals not humans).sh.
6	((comment or editorial or meta-analysis or practice-guideline or review or letter or journal correspondence) not "randomized controlled trial").pt.
7	(random sampl$ or random digit$ or random effect$ or random survey or random regression).ti,ab. not "randomized controlled trial".pt.
8	4 not (5 or 6 or 7)
9	(chronic obstructive lung disease or COPD or chronic obstructive pulmonary disease or chronic bronchitis or emphysema or COAD or chronic obstructive airway disease).ti,ab.
10	(single triple or long acting beta-2 agonist or LABA or long acting antimuscarinic or LAMA or inhaled corticosteroid or ICS or beclomethasone dipropionate/formoterol fumarate/glycopyrronium bromide or budesonide/glycopyrronium bromide/formoterol fumarate or fluticasone furoate/umeclidinium/vilanterol).ti,ab.
11	(single dual or long acting beta-2 agonist or LABA or long acting antimuscarinic or LAMA or inhaled corticosteroid or ICS or beclomethasone dipropionate or formoterol fumarate or glycopyrronium bromide or budesonide or fluticasone furoate or umeclidinium or indacaterol or vilanterol).ti,ab.
12	(death or AECOPD or Exacerbations or hospitalizations or St George s Respiratory Questionnaire score or SGRQ score or transitional dyspnoea index or TDI or forced expiratory volume in one second or FEV1 or adverse events).ti,ab.
13	8 and 9 and 10 and 11 and 12
